# Sex-Differences in the Oxygenation Levels of Intercostal and Vastus Lateralis Muscles During Incremental Exercise

**DOI:** 10.3389/fphys.2021.738063

**Published:** 2021-10-01

**Authors:** Maximiliano Espinosa-Ramírez, Eduardo Moya-Gallardo, Felipe Araya-Román, Santiago Riquelme-Sánchez, Guido Rodriguez-García, W. Darlene Reid, Ginés Viscor, Oscar F. Araneda, Luigi Gabrielli, Felipe Contreras-Briceño

**Affiliations:** ^1^Laboratory of Exercise Physiology, Department of Health Science, Faculty of Medicine, Pontificia Universidad Católica de Chile, Santiago, Chile; ^2^Physical Therapy, Interdepartmental Division of Critical Care Medicine, University of Toronto, Toronto, ON, Canada; ^3^KITE Research Institute, Toronto Rehabilitation Institute, University Health Network, Toronto, ON, Canada; ^4^Physiology Section, Department of Cell Biology, Physiology and Immunology, Faculty of Biology, Universitat de Barcelona, Barcelona, Spain; ^5^Laboratory of Integrative Physiology of Biomechanics and Physiology of Effort (LIBFE), Faculty of Medicine, Kinesiology School, Universidad de los Andes, Santiago, Chile; ^6^Division of Cardiovascular Diseases, Faculty of Medicine, Advanced Center for Chronic Diseases (ACCDiS), Pontificia Universidad Católica de Chile, Santiago, Chile

**Keywords:** cost of breathing, exercise, near-infrared spectroscopy, respiratory muscles, sex

## Abstract

This study aimed to examine sex differences in oxygen saturation in respiratory (SmO_2_-*m.intercostales*) and locomotor muscles (SmO_2_-*m.vastus lateralis*) while performing physical exercise. Twenty-five (12 women) healthy and physically active participants were evaluated during an incremental test with a cycle ergometer, while ventilatory variables [lung ventilation ($\dot{\text{V}}$E), tidal volume (Vt), and respiratory rate (RR)] were acquired through the breath-by-breath method. SmO_2_ was acquired using the MOXY^®^ devices on the *m.intercostales* and *m.vastus lateralis*. A two-way ANOVA (sex × time) indicated that women showed a greater significant decrease of SmO_2_-*m.intercostales*, and men showed a greater significant decrease of SmO_2_-*m.vastus lateralis*. Additionally, women reached a higher level of ΔSmO_2_-*m.intercostales* normalized to $\dot{\text{V}}$E (L⋅min^–1^) (*p* < 0.001), whereas men had a higher level of ΔSmO_2_-*m.vastus lateralis* normalized to peak workload-to-weight (watts⋅kg^–1^, PtW) (*p* = 0.049), as confirmed by Student’s *t*-test. During an incremental physical exercise, women experienced a greater cost of breathing, reflected by greater deoxygenation of the respiratory muscles, whereas men had a higher peripheral load, indicated by greater deoxygenation of the locomotor muscles.

## Introduction

Physiological sex-related differences during dynamic physical exercise have been a subject of recent interest. A primary difference is the greater oxygen consumption of the respiratory muscles ($\dot{\text{V}}$O_2–*RM*_), such as the *m.intercostales*, in women (13.8 vs. 9.4% in men) (Lomauro and Aliverti, 2018), requiring higher lung ventilation ($\dot{\text{V}}$E) in response to metabolic demands (Guenette et al., 2009; Welch et al., 2018). This greater $\dot{\text{V}}$O_2–*RM*_ is attributed to increased resistance and elasticity load of the respiratory system, consequent to a smaller rib cage (Bellemare et al., 2003), a mechanical disadvantage of *m.diaphragma*, lower airway diameter (Lomauro and Aliverti, 2018) and higher recruitment of the accessory respiratory muscles (Sheel et al., 2004; Mitchell et al., 2018). The increase of $\dot{\text{V}}$O_2–*RM*_ requires increased blood flow to the respiratory muscles, which may restrict nutrient and oxygen supply to peripheral exercising muscles, thereby limiting the continued performance of exercise (metareflex) (Guenette and Sheel, 2007; Dominelli et al., 2017).

The oxygen saturation of muscles (SmO_2_) reflects the supply and demand of oxygen in this tissue. This can be determined using continuous-wave near-infrared spectroscopy (NIRS, 630–850 nm) that measures changes in oxygenated hemoglobin (O_2_ Hb) and myoglobin (mHb) at a microvascular level (Perrey and Ferrari, 2018; Alvares et al., 2020). A previous study reported high reliability of the measurement protocol of SmO_2_ in *m.intercostales* and *m.vastus lateralis* during maximal incremental treadmill exercise in male long-distance runners (Contreras-Briceño et al., 2019). However, investigations of sex-related differences and how SmO_2_ levels change in these muscles during exercise in women is scarce. Therefore, potential sex-related differences need to be explored further to define their impact on sports performance and in rehabilitation.

This study aimed to evaluate the sex differences of SmO_2_ in *m.intercostales* and *m.vastus lateralis* during an incremental cycle test until exhaustion and to analyze the changes in SmO_2_ in relative to ventilatory variables, peripheral workload, and aerobic capacity ($\dot{\text{V}}$O_2–*peak*_). To our knowledge, this is the first study that evaluates sex-related differences of SmO_2_ in the *m.intercostales* and *m.vastus lateralis* in highly recruited muscles of healthy subjects while performing an incremental maximal exercise.

## Materials and Methods

### Participants

Twenty-five physically active participants (12 women) were assessed. Anthropometric and pulmonary functional characteristics of the subjects are shown in Table 1. The participants reported no history of respiratory, cardiovascular, metabolic, musculoskeletal, neoplastic diseases or acute infections for at least 2 weeks before testing. They did not take anti-inflammatory medications, illicit drugs, antioxidants, or any other dietary supplements. All participants were thoroughly informed (in verbal and written forms) of the study procedures, and all of them signed informed consent forms. This study followed the Declaration of Helsinki (Harriss et al., 2017) and was approved by the ethics committee of the Faculty of Medicine of Pontificia Universidad Católica of Chile (project no. 19042213).

**TABLE 1:** Participants’ characteristics.

	**Men (*n* = 13)**	**Women (*n* = 12)**	***p-*value**	**[95% CI]**
Age (years)	22 ± 1	21 ± 1	0.461	[−1.3 to 0.6]
Height (cm)	175 ± 1*	162 ± 2	***<0.001***	[−16.5 to −10.26]
Weight (kg)	68 ± 6*	55 ± 3	***<0.001***	[−17.5 to −8.6]
BMI (kg⋅m^2^)	22.0 ± 1.3*	20.9 ± 1.0	***0.023***	[−2.1 to −0.1]
FVC (L)	5.5 ± 0.3*	3.8 ± 0.4	***<0.001***	[−1.9 to −1.3]
FEV_1_ (L)	4.5 ± 0.2*	3.4 ± 0.4	***<0.001***	[−1.3 to −0.8]
FEV_1_⋅FVC^–^^1^ (%)	81.2 ± 3.4	89.1 ± 9.7*	***0.012***	[1.8 to 13.9]
MIP (cm H_2_O)	150 ± 27	132 ± 30	0.137	[−41.1 to 6.0]
TIRE (s)	730 ± 84	823 ± 68	0.060	[−4.4 to 192.3]

Data are presented as means ± standard deviations, with 95% confidence intervals. *p < 0.05 (statistical difference between groups, according to the Student‘s t-test).

*BMI, body mass index; FVC, forced vital capacity; FEV_1_, forced expiratory volume in the first second; MIP, maximal inspiratory pressure; TIRE, test of incremental respiratory endurance. Statistically significant differences (p < 0.05) are marked in bold.*

### Protocol

The participants were evaluated at the Laboratory of Exercise Physiology of Pontificia Universidad Católica of Chile during two sessions, separated by an interval of 24 h interval. All procedures were performed under laboratory environmental conditions (temperature, 22 ± 2°C; relative humidity, 40 ± 2%) and within a similar time frame (9:00 a.m.–2:00 p.m.). Participants were asked to avoid physical activities for 24 h before the measurements and to avoid alcohol, caffeine, and other stimulants and food for at least 3 h prior to the evaluations.

#### First Session

Anthropometric evaluations were measured (weight, height, and body mass index). Subsequently, spirometry (Microlab, model ML3500, CareFusion^®^, San Diego, United States) was performed according to the American Thoracic Society (ATS)⋅European Respiratory Society (ERS) protocol (Gibson et al., 2002), utilizing the reference values of Knudson et al. (1983). Finally, maximal inspiratory pressures (MIP) were evaluated using a pneumometer (Micro MRC, CareFusion^®^, Traunstein, Germany) according to the protocol proposed by the American Thoracic Society (ATS) and the European Respiratory Society (Gibson et al., 2002), utilizing the reference values of Black and Hyatt (Hyatt and Black, 1969). Respiratory resistance was also measured using the *Test of Incremental Respiratory Endurance* (TIRE) with *POWERbreathe*^®^ threshold loading devices (IMT Technologies Ltd., Birmingham, United Kingdom) according to the modified protocol of Cahalin and Arena (2015), which consisted of a series of 90 s at a respiratory rate (RR) of 30 breaths⋅min^–1^, with an initial load of 10% of the MIP and increased by 10% until task failure or inability to maintain the RR.

#### Second Session

##### Peak oxygen consumption test ($\dot{\text{V}}$O_2–*peak*_)

Peak oxygen consumption ($\dot{\text{V}}$O_2–*peak*_) was evaluated using an ergospirometer (MasterScreen CPX, Jaeger^®^, Germany) via the breath-by-breath method during an incremental cycle ergometer protocol (ViaSprint 150P, Ergoline GmH, Traunstein, Germany). The protocol consisted of a 1-min rest, 5-min warm-up period at 40 watts, followed by an increase of 20 watts every 2 min until exhaustion, despite standardized verbal stimuli (respiratory quotient, 1.20 ± 0.05) (see Supplementary Material I; Contreras-Briceño et al., 2020). The participants were requested to maintain a cadence between 70 and 90 rpm. The $\dot{\text{V}}$O_2–*peak*_ was calculated as the highest value obtained during the last 30 s of the incremental test, despite increasing the exercise intensity (<150 ml⋅min^–1^ of exercise) (Day et al., 2003). A cool down of 4 min of submaximal exercise was performed before allowing the patients to rest. At baseline and throughout the test, the heart rate, pulse oxygen saturation, and blood pressure were measured. Before every test, the gas analyzer was calibrated according to the instructions provided by the manufacturer.

##### Measurement of muscle oxygenation (SmO_2_)

**FIGURE 1:**
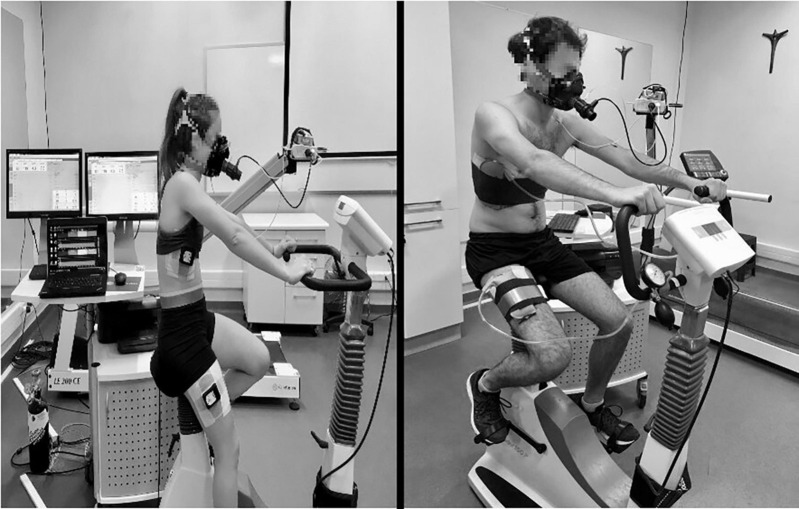
The participant’s position on the cycle ergometer.

The saturation of oxygen in muscles (SmO_2_) was evaluated using continuous-wave near-infrared spectroscopy (NIRS, 630–850 nm), a non-invasive method (MOXY^®^, Fortiori, Desing LLC, Minnesota, United States). This device measures the absorbance of infrared light by oxygenated (O_2_ Hb), deoxygenated hemoglobin (HHb), and myoglobin (mHb) at a microvascular level (Austin et al., 2005). From these values, SmO_2_ was calculated using PeriPedal (PeriPedal^®^, IN, United States) at a sampling frequency of 2 Hz (McManus et al., 2018) from the *m.intercostales* (SmO_2_-*m.intercostales*) and *m.vastus lateralis* (SmO_2_-*m.vastus lateralis*), according to a previous protocol (Contreras-Briceño et al., 2019). In brief, for *m.intercostales*, a MOXY^®^ device was placed on the seventh intercostal space at the anterior axillary line in the right thoracic area. To determine the level of SmO_2_ in the locomotor muscles, a second MOXY^®^ device was placed over the *m.vastus lateralis*, 5 cm lateral to the midline of the thigh and landmarked midway between the upper edge of the patella and the greater trochanter of the right femur. The devices were fixed to the skin with double-sided sticky tape and hypoallergenic skin tape (see Figure 1).

### Data Analysis

Each participant had an initial record of 90 s on the cycle ergometer, followed by 60 s of a baseline resting phase, during which data acquisition began was synchronized. For data analysis, data were compared across percentages of the test duration (0–100% of task), with the time of $\dot{\text{V}}$O_2–*peak*_ set as 100%. To complement the analysis, a triphasic model of exercise intensity was used, where ventilatory thresholds (VT1 and VT2) were calculated by two expert researchers using a visual method (Guazzi et al., 2012). An opinion of a third expert was considered in cases of discrepancy during the analysis (Vainshelboim et al., 2017). The values used for ventilatory variables [$\dot{\text{V}}$E, RR, and tidal volume (Vt)] and SmO_2_ were obtained from the average of the last 30 s during each phase. At the end of the protocol, the exhaustion of the participants was evaluated using the Borg Modified Rating Perceived Scale (RPE) (see Supplementary Material I).

To compare differences of muscle oxygen levels between men and women, data are reported as: (i) maximum changes or differences (Δ), (e.g., ΔSmO_2_-*m.intercostales* = SmO_2_-*m.intercostales* rest—SmO_2_-*m.intercostales* peak phase) and (ii) percentage of change, dividing the SmO_2_ value of each phase by the SmO_2_ of rest phase (%-change), (e.g., SmO_2_-*m.intercostales* (%-change) = (SmO_2_-*m.intercostales* phase⋅SmO_2_-*m.intercostales* rest phase^–1^). In addition, ΔSmO_2_-*m.intercostales* was normalized by Δ$\dot{\text{V}}$E (SmO_2_-*m.intercostales* ⋅Δ$\dot{\text{V}}$E^–1^) and the ΔSmO_2_-*m.vastus lateralis* was normalized to peak workload-to-weight (watts⋅kg^–1^) (SmO_2_-*m.vastus lateralis*⋅ PtW^–1^).

### Statistical Analysis

Normality of the data was evaluated using the Shapiro-Wilk test. The descriptive characteristics of the participants were compared using the Student’s *t*-test. The differences between sexes with regard to SmO_2_*-m.intercostales*, SmO_2_-*m.vastus lateralis*, SmO_2_ *m.intercostales—m.vastus lateralis* ratio, and ventilatory variables ($\dot{\text{V}}$E, RR, and Vt), which are expressed according to the % task, were analyzed using the two-way ANOVA test, reporting differences between conditions when the interaction of the factors (sex × time) was significant (*p <* 0.05). Subsequent multiple comparisons were analyzed used the Sidak *post-hoc* test. Comparisons of ΔSmO_2_-*m.intercostales*⋅Δ$\dot{\text{V}}$E^–1^ and ΔSmO_2_-*m.vastus lateralis*⋅PtW^–1^ were performed using the Student’s *t*-test. The correlation between ΔSmO_2_-*m.vastus lateralis* (%) and ventilatory variables were assessed using Pearson’s correlation coefficient. Statistical significance was set at *p <* 0.05. The statistical analysis was performed using the GraphPad Prism (version 8.0; San Diego, California, United States).

## Results

### Muscle Oxygenation

#### SmO_2_-*m.intercostales* (%)

**FIGURE 2:**
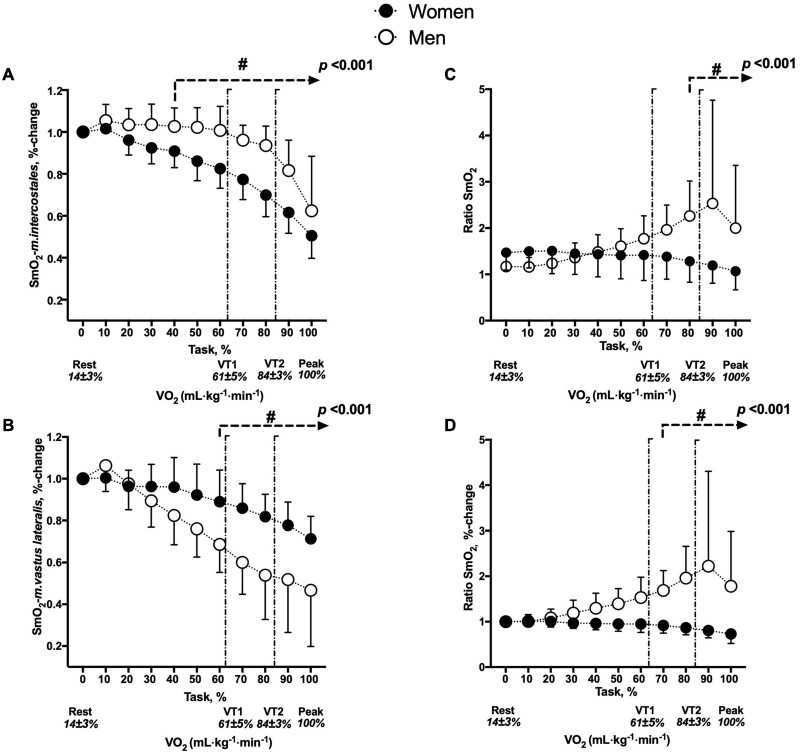
Changes in SmO_2_ between men and women. **(A)** SmO_2_-*m.intercostales* (%-change), **(B)** SmO_2_-*m.vastus lateralis* (%-change), **(C)** SmO_2_ ratio (absolute values) and **(D)** SmO_2_ ratio (%-change). ^#^*p* < 0.05: comparison between sex with respect to percentage of task. The two vertical lines represent ventilatory thresholds (VT1, 61 ± 5% to VO_2–*peak*_ and VT2, 84% ± 3% to VO_2–*peak*_). The interaction between factors (sex × time) is represented by the *p-*value.

The interaction of factors (sex × time) was different in the SmO_2_-*m.intercostales* expressed as a percentage of the performed task (*p <* 0.001). The SmO_2_-*m.intercostales* (% change) decreased more significantly in women than in men (between 40 and 100%, *p <* 0.001) and between VT1 and VT2 (*p <* 0.001) (see Figure 2A). The ΔSmO_2_-*m.intercostales* was greater in women than in men (39 ± 9 vs. 27 ± 19, respectively, *p* = 0.048) (see Supplementary Material I).

#### SmO_2_-*m.vastus lateralis* (%)

The interaction of factors (sex × time) was different in the SmO_2_-*m.vastus lateralis* expressed as a percentage of the performed task (*p <* 0.001). The SmO_2_-*m.vastus lateralis* (% change) decreased more significantly in men than in women (between 60 and 100%, *p* < 0.001) and between VT1, VT2, and $\dot{\text{V}}$O_2–*peak*_ phases (*p* < 0.001) (see Figure 2B). The ΔSmO_2_-*m.vastus lateralis* was greater in men than in women (34 ± 18 vs. 16 ± 9, respectively, *p* < 0.001) (see Supplementary Material I).

#### SmO_2_ Ratio

The interaction of factors (sex × time) was different in the SmO_2_ ratio (absolute values) expressed as a percentage of the performed task (*p* < 0.001) and the SmO_2_ ratio (%-change) expressed as a percentage of the performed task (*p* < 0.001). In terms of absolute values, the decrease was more in women than in men (between 80 and 100%, *p* < 0.001) and in VT2 and $\dot{\text{V}}$O_2–*peak*_ phases (*p* < 0.001) (see Figure 2C). In terms of %-change, the decrease was more in women than in men (between 70 and 100%, *p* < 0.001) and in VT2 and $\dot{\text{V}}$O_2–*peak*_ phases (*p* < 0.001) (see Figure 2D).

#### Total Hemoglobin (g⋅dL^–1^)

There were no differences in THb in the *m.intercostales* and *m.vastus lateralis* in both groups (see Supplementary Material I).

### Peak Oxygen Consumption Test ($\dot{\text{V}}$O_2–*peak*_)

**FIGURE 3:**
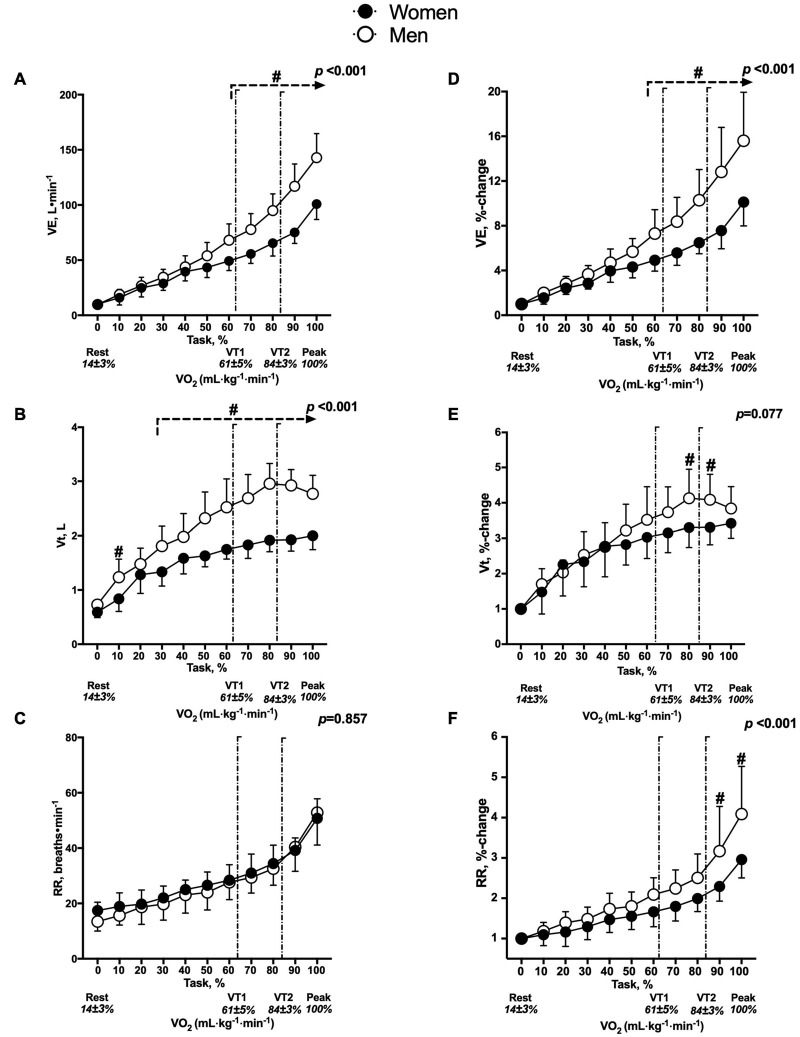
Changes in ventilatory variables between men and women. The panels on the right indicate absolute values, **(A)** $\dot{\text{V}}$E (L⋅min^–^^1^), **(B)** Vt (L) and **(C)** RR (breaths⋅min^–1^). The panels on the left indicate the percentage to rest, **(D)** $\dot{\text{V}}$E (%-change), **(E)** Vt (%-change) and **(F)** RR (%-change). ^#^*p* < 0.05: comparison between sex with respect to percentage of task. The two vertical lines represent the ventilatory thresholds (VT1, 61 ± 5% to VO_2–*peak*_ and VT2, 84 ± 3% to VO_2–*peak*_). The interaction between factors (sex × time) is represented by the *p-*value.

Compared to women, men had higher absolute (3470 ± 436 ml⋅min^–1^ vs. 2156 ± 189 ml⋅min^–1^, respectively, *p <* 0.001) and relative (51.0 ± 5.3 ml⋅kg⋅min^–1^ vs. 39.3 ± 3.0 ml⋅kg⋅min^–1^, respectively, *p* < 0.001) $\dot{\text{V}}$O_2–*peak*_ values. Regarding the workload (watts), men demonstrated greater values in absolute terms (256 ± 23 vs. 164 ± 17, *p* < 0.001) and when normalized to body weight (PtW) (3.8 ± 0.4 vs. 3.0 ± 0.3, *p* < 0.001). Regarding the ventilatory variables, men reached greater $\dot{\text{V}}$E_*max*_ expressed in absolute terms (L⋅min^–1^) (142.9 ± 21.6 vs. 98.9 ± 14.9, *p* < 0.001) and in %-change (15.6 ± 4.3 vs. 9.9 ± 2.2, *p* = 0.037) (see Supplementary Material I). The interaction of factors (sex × time) was different in the $\dot{\text{V}}$E (L⋅min^–1^) (*p* < 0.001) and $\dot{\text{V}}$E (%-change) (*p* < 0.001). When expressing this variable regarding the % task in absolute terms (see Figure 3A) and %-change, men reached a greater value from 60 to 100% of the protocol (*p* < 0.001) (see Figure 3B). Concerning the RR_*max*_ expressed in absolute terms (bpm), there were no differences between sexes (*p* = 0.457). The interaction of factors (sex × time) was not different in the RR (breaths⋅min^–1^) (*p* = 0.857), but RR (%-change) was different (*p* < 0.001). The level of RR (%-change) in the peak phase was greater in men than in women (4.1 ± 1.2 vs. 2.9 ± 0.4, *p* < 0.001). When expressing this variable in relation to the % task, men reached a greater value from 90 to 100% of the protocol (*p* < 0.001) (see Figure 3F). The level of Vt_*max*_ expressed in absolute terms (L) was greater in men than in women (2.9 ± 0.3 vs. 2.0 ± 0.3, respectively, *p <* 0.010). The interaction of factors (sex × time) was different in the Vt (L) (*p* < 0.001), but not in the Vt (%-change) (*p* = 0.070). When expressing absolute Vt_*max*_ in relation to the % task, it was greater in men (between 30 and 100% during the protocol, *p* < 0.001) (see Figure 3B). Regarding the Vt (%-change), the value was greater in men (between 80 and 90% of the test, *p* < 0.027) (see Figure 3E).

The variables mentioned above that consider the triphasic model of exercise intensity are presented in Supplementary Material I.

#### SmO_2_ in Relation to Lung Ventilation and Peak Workload-to-Weight

**FIGURE 4:**
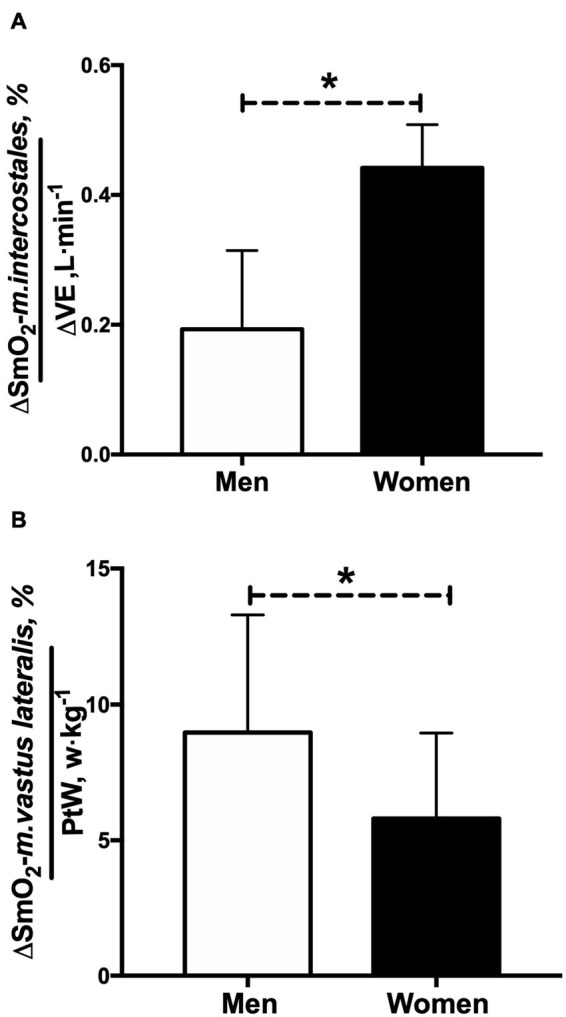
Comparison in SmO_2_ between men and women standardized by **(A)** Δ$\dot{\text{V}}$E ($\dot{\text{V}}$E peak—$\dot{\text{V}}$E rest phase, ΔSmO_2_-*m.intercostales*⋅Δ$\dot{\text{V}}$E^–^^1^) and **(B)** PtW (peak workload-to-weight, ΔSmO_2_-*m.vastus lateralis*⋅PtW^–1^). *p < 0.05.

The ΔSmO_2_-*m.intercostales*⋅$\dot{\Delta \text{V}}$E^–^^1^ was greater in women than in men (0.5 ± 0.1 vs. 0.2 ± 0.1, respectively, *p* < 0.001) (see Figure 4A), while the ΔSmO_2_-*m.vastus lateralis*⋅PtW^–1^ was greater in men than in women (10.1 ± 4.8 vs. 7.2 ± 4.0, respectively, *p* = 0.049) (see Figure 4B).

#### Correlations

**FIGURE 5:**
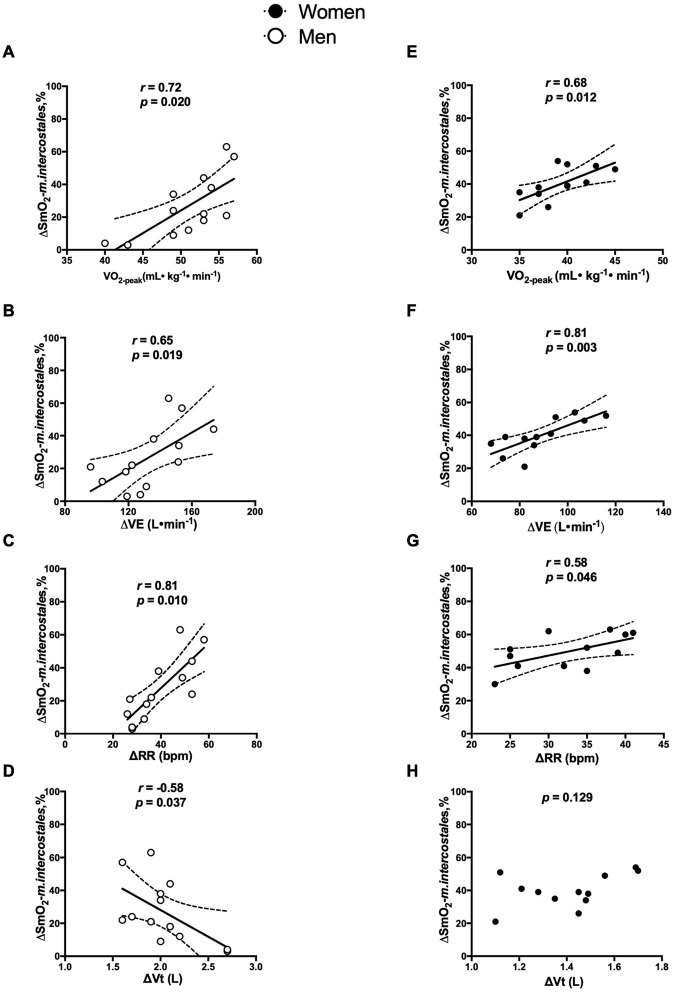
Correlation in men and women between ΔSmO_2_-*m.intercostales* with: **(A,E)** $\dot{\text{V}}$O_2–*peak*_(ml⋅kg^–1^⋅min^–1^), **(B,F)** $\Delta \dot{\text{V}}$E ($\dot{\text{V}}$E peak—$\dot{\text{V}}$E rest phase, L⋅min^–1^), **(C,G)** RR (RR peak – RR rest phase, breaths⋅min^–1^) and **(D,H)** Vt (Vt peak – Vt rest phase, L).

In men, ΔSmO_2_-*m.intercostales* was directly associated with $\dot{\text{V}}$O_2–*peak*_ (*r* = 0.72, *p* = 0.020) (see Figure 5A), Δ$\dot{\text{V}}$E (*r* = 0.65, *p* = 0.019) (see Figure 5B), and ΔRR (*r* = 0.80, *p* < 0.010) (see Figure 5C), and inversely associated with ΔVt (*r* = ^–^0.58, *p* = 0.035) (see Figure 5D). In women, ΔSmO_2_-*m.intercostales* was associated with $\dot{\text{V}}$O_2–*peak*_ (*r* = 0.68, *p* = 0.012) (see Figure 5E), Δ$\dot{\text{V}}$E (*r* = 0.81, *p* = 0.003) (see Figure 5F) and ΔRR (*r* = 0.58, *p* = 0.046) (see Figure 5G), but not with ΔVt (*p* = 0.129) (see Figure 5H).

## Discussion

Our main findings are that women have a greater decrease in SmO_2_-*m.intercostales*, in spite of achieving a lower maximal change in lung ventilation, and that men experience a consistent decrease in the SmO_2_-*m.vastus lateralis* induced by a greater peripheral workload.

### Sex Differences in SmO_2_-*m.intercostales*

Women show a greater decrease of SmO_2_-*m.intercostales* (%-change) from 40 to 100% of the maximal exercise test, possibly attributed to the great relative recruitment of the respiratory musculature, required to reach the $\dot{\text{V}}$E level to meet the exercise metabolic demands (Contreras-Briceño et al., 2021). This is consistent with previous findings that reported that the contractile activity of accessory respiratory muscles (*m.sternocleidomastoideus* and *m.scalenus*) was greater in women during a bicycle endurance test at a workload of 85%. This is attributed to their thoracic and pulmonary characteristics and dependence on thoracic and accessory respiratory muscles during intense exercise has been postulated to be an adaptation that diminishes the risk of diaphragmatic fatigue (Mitchell et al., 2018). Women have increased resistive respiratory (WOB_*R*_) and elastic (WOB_*e*_) work, lower forced vital capacity, and high limited expiratory flow (Sheel and Guenette, 2008; Sheel and Romer, 2012). A similar study reported that this greater level of WOB increases the respiratory oxygen consumption in women during maximal exercise (13.8 vs. 9.4% in men) (Lomauro and Aliverti, 2018). Our findings are aligned with these data, as we found a greater ΔSmO_2_-*m.intercostales* in women than in men (39% ± 9% vs. 27% ± 19%, respectively) (see Supplementary Material I).

In both groups, the participants who achieved greater $\dot{\text{V}}$E values induced greater deoxygenation in *m.intercostales*, which is consistent with the finding of a previous study (Contreras-Briceño et al., 2019). Interestingly, even though women achieved a lower $\dot{\Delta \text{V}}$E than men (88.7 ± 14.5 vs. 133.1 ± 21.8 L⋅min^–1^, respectively), they induced a higher level of ΔSmO_2_-*m.intercostales* normalized by $\Delta \dot{\text{V}}$E; this is consistent with a previous study that reported women reaching a higher level of $\dot{\text{V}}$O_2–*RM*_ in spite of only achieving a $\dot{\text{V}}$E that was 25% of men’s values (Lomauro and Aliverti, 2018). Another related observation was that participants who demonstrated a greater decrease of ΔSmO_2_-*m.intercostales* also showed a larger change in RR, for which may be an effect of ventilatory strategy during exercise.

Considering the triphasic model of exercise intensity proposed by Skinner et al. (Skinner and McLellan, 1980), the SmO_2_-*m.intercostales* showed early changes that were lower in women at VT1 (0.8 ± 0.1 vs. 1.0 ± 0.1 in men) and VT2 (0.6 ± 0.4 vs. 0.8 ± 0.1 in men). Similarly, in women, exercise intensities on VT1 lead to a greater level of oxygen consumption by the respiratory muscles ($\dot{\text{V}}$O_2–*RM*_), a phenomenon recognized as a limiting factor in physical performance progression in women (Guenette and Sheel, 2007). However, there are no differences between sexes at the peak exercise phase, which could be explained by the exponential increase of $\dot{\text{V}}$E at the expense of RR (Wanke et al., 1991).

### Sex Differences in SmO_2_-*m.vastus lateralis*

Our results show that men have greater decreases in SmO_2_-*m.vastus lateralis* from 60 to 100% of the workload, possibly because of a greater contraction speed and cross-sectional area in locomotor muscles, leading to a sustained high intensity of exercise requiring a higher level of oxygen consumption level in peripheral muscles (Hakkinen, 1993; Fulco et al., 1999). In addition, as expected, men reached a higher absolute peripheral workload (256 vs. 164 watts in women) and PtW (3.8 vs. 3.0 watts⋅kg^–1^ in women), which is in agreement with previous reports, wherein it was found that men perform more peripheral muscle work that is supported by a higher neuromuscular activity and IIx fiber recruitment (Miller et al., 1993). Thus, our study found that greater decreased ΔSmO_2_-*m.vastus lateralis* normalized by relative peripheral workload was higher in men (see Figure 4B). Further, women have a larger fiber I ratio and a higher capillary density in peripheral muscles, which provides better oxygen supply allowing them to sustain muscle work with a higher oxygen consumption capacity. Such related muscle mass and fiber type characteristics are worthy of investigation in future studies (Simoneau and Bouchard, 1989; Glenmark et al., 2004).

Another aspect to discuss is the length of time of exercise protocol used. The literature suggests that peak oxygen consumption tests should not exceed 10 min of duration to limit muscle fatigue as a cause of exercise cessation (Buchfuhrer et al., 1983). However, this finding has been questioned by Midgley et al. (2008) who showed that longer test generates higher $\dot{\text{V}}$O_2–*peak*_ values, suggesting that the cycloergometer tests should last between 7 and 26 min (Midgley et al., 2008). In this sense, incremental exercises by 20 watts⋅min^–1^ had been associated with progressive deoxygenation in the *m.vastus lateralis*, *m.rectus femoris*, and *m.vastus medialis*, similarly to our findings (Chin et al., 2011). Consequently, utilization of this time-stage protocol can induce higher power and total work than ramp tests (Zuniga et al., 2012). Based on these previous findings, we used an exercise protocol with a longer 2-min incremental stage, without discarding that the fiber recruitments associated with exercising could affect the results obtained; this is a relevant aspect to elucidate in future research.

### Sex Differences in the SmO_2_ Ratio

Synchronous evaluation of both muscle groups determined the SmO_2_ ratio by calculating the preponderance of the muscle group with a higher oxygen demand during the study protocol. In this regard, we found that women had fewer changes than men (absolute, between 80 and 100%; relative, from 70 to 100%) (see Figures 3C,D). These findings could be explained by the increase in the cost of breathing, which leads to a higher blood flow to respiratory muscles, probably restricting oxygen supply to the locomotor muscles (metabolic reflex) (Boushel, 2010). Moreover, women reach a higher level of $\dot{\text{V}}$O_2–*RM*_ because of the increase in $\dot{\text{V}}$E, which limits oxygen supply to the locomotor muscles (Dominelli et al., 2015, 2017), also consistent with the study by Guenette et al. (2007). Nonetheless, other authors have reported a homogeneous distribution of blood flow in women, resulting from a lower sympathetic stimulation and vascular resistance in lower extremities, which does not negatively impact exercise progression (Smith et al., 2016). These conflicting data require further examination that would include evaluation methods to clarify of the effect of metabolic reflex on sports performance in women.

### Limitations

By device limitations, it was not possible to completely assess the respiratory and locomotor muscles in a single test. The lack of adipose thickness measures by skinfold and/or ultrasonography reports limits verification that the muscle tissue was within the measuring range of the MOXY device (12.5 mm) (McManus et al., 2018). However, our participants were at the lower end of the normal BMI range. In addition, cyclic hormonal variations should be considered in women participants, given that edema, dehydration, and altered thermoregulation are factors affecting physical performance by modifying the ventilatory center response (Kilbride et al., 2003); this is an aspect that requires further study. Future studies could consider the stage of the menstrual cycle; however, menstrual cycle is not consistently found to influence exercise performance (McNulty et al., 2020). Recently, it was reported that 36% of high-performance female athletes stated that their menstrual cycle impacted negatively on their performance for at least some or most of the time (Heather et al., 2021). In contrast, it did not affect the production of muscle strength and power (McNulty et al., 2020; Dasa et al., 2021). Furthermore, we recommend to future research could consider the confounding influence of physical fitness levels of participants.

## Conclusion

This study compared the differences in oxygen saturation (SmO_2_) of *m.intercostales* and *m.vastus lateralis* between men and women during maximal physical exercise. Our main findings indicate that women show a greater decrease in SmO_2_-*m.intercostales*, which could be attributed to anatomical and functional difference of their lungs and thoraces. In contrast, men reach a greater decrease in SmO_2_-*m.vastus lateralis*, which is associated with a higher peripheral workload. These results provide new knowledge on the primary muscle groups recruited during maximal effort and therefore shed light on the sex-differences between subjects of similar physical condition. In future studies, we suggest studying the effects of different respiratory muscle training methods in SmO_2_-*m.intercostales* during physical exercise and their impact on sports performance. Consequently, optimal guidelines for adequate training protocols could be formulated, for athletes and clinical settings.

## Data Availability Statement

The original contributions presented in the study are included in the article/Supplementary Material, further inquiries can be directed to the corresponding author/s.

## Ethics Statement

This study was reviewed and approved by the Ethics Committee of the Faculty of Medicine of Pontificia Universidad Católica of Chile (Project no. 19042213). The participants provided their written informed consent to participate in this study. Written informed consent was obtained from the individual(s) for the publication of any potentially identifiable images or data included in this article.

## Author Contributions

ME-R, GV, OA, LG, and FC-B substantially contributed to the concept and design of the study. WR and EM-G contributed to the assay setup. ME-R, FA-R, SR-S, GR-G, and FC-B contributed to data acquisition, analysis, and interpretation. ME-R, WR, GV, OA, LG, and FC-B contributed to the data interpretation, discussion, and manuscript preparation. ME-R, EM-G, and FC-B wrote the manuscript. All authors contributed to and critically revised the manuscript for important intellectual content and approved the submitted version.

## Conflict of Interest

The authors declare that the research was conducted in the absence of any commercial or financial relationships that could be construed as a potential conflict of interest.

## Publisher’s Note

All claims expressed in this article are solely those of the authors and do not necessarily represent those of their affiliated organizations, or those of the publisher, the editors and the reviewers. Any product that may be evaluated in this article, or claim that may be made by its manufacturer, is not guaranteed or endorsed by the publisher.
