# Supplementary material for: Sex-Differences in the Oxygenation Levels of Intercostal and Vastus Lateralis Muscles During Incremental Exercise
Source: Front Physiol. 2021 Oct 1;12:738063. doi: 10.3389/fphys.2021.738063 (PMC8517227; doi:10.3389/fphys.2021.738063)
Supplement: Supplementary file 1 [file Data_Sheet_1.docx]

**Supplementary material I.** Variables obtained at peak oxygen consumption test.

|  |  | Men (n=13) | |  |  |  | Women (n=12) | |  |
| --- | --- | --- | --- | --- | --- | --- | --- | --- | --- |
|  | Rest | VT1 | VT2 | Peak |  | Rest | VT1 | VT2 | Peak |
| Workload (watts) | - | 146±9**^#^** | 225±22**^#^** | 256±23**^#^** |  | - | 95±16 | 141±17 | 164±17 |
| WtW (watts·kg^-1^) | 0.4±0.1 | 2.2±0.2**^#^** | 3.3±0.3**^#^** | 3.8±0.4**^#^** |  | 0.5±0.3 | 1.7±0.3 | 2.6±0.3 | 3.0±0.3 |
| $\dot{V}$O_2_ (ml·min ^-1^) | 407±67 | 2053±188**^#^** | 2887±315**^#^** | 3469±436**^#^** |  | 351±51 | 1329±145 | 1835±165 | 2156±189 |
| $\dot{V}$O_2_ (ml·kg^-1^·min^-1^) | 5.9±0.7 | 30.4±3.8**^#^** | 42.5±3.6**^#^** | 51.0±5.1**^#^** |  | 6.4±0.9 | 24.3±2.9 | 33.4±0.7 | 39.3±3.0 |
| Respiratory quotient | 0,9±0.07 | 0,99±0,05 | 1,10±0,06 | 1,20±0,05 |  | 0,8±0.07 | 0,98±0,07 | 1,10±0,02 | 1,20±0,05 |
| Heart rate (Bpm) | 79±9 | 147±13 | 175±13 | 185±6 |  | 88±9 | 155±12 | 175±12 | 183±9 |
| Respiratory rate (bpm) | 13±3 | 23±6 | 37±7 | 53±11 |  | 17±3 | 25±4 | 34±7 | 50±8 |
| Respiratory rate (%-change) | 1.0 | 1.7±0.3 | 2.9±0.7**^#^** | 4.1±1.2**^#^** |  | 1.0 | 1.5±0.3 | 1.9±0.4 | 2.9±0.4 |
| Tidal volume (L) | 0.7±0.1 | 2.6±0.4**^#^** | 2.9±0.3**^#^** | 2.8±0.3**^#^** |  | 0.5±0.1 | 1.7±0.2 | 1.9±0.3 | 2.0±0.3 |
| Tidal volume (%-change) | 1.0 | 3.6±0.8 | 4.0±0.6**^#^** | 3.8±0.6 |  | 1.0 | 3.1±0.7 | 3.4±0.5 | 3.4±0.4 |
| Lung ventilation (L·min^-1^) | 10±3.4 | 56.8±8.9**^#^** | 104.8±21.6**^#^** | 142.9±21.6**^#^** |  | 10.2±2.0 | 43.6±5.2 | 66.7±12.2 | 98.9±14.9 |
| Lung ventilation (%-change) | 1.0 | 6.1±1.3 | 11.4±3.1**^#^** | 15.6±4.3**^#^** |  | 1.0 | 4.4±0.9 | 6.7±1.4 | 9.9±2.2 |
| Borg Modified Rating Perceived Scale | - | - | - | 9±1 |  | - | - | - | 9±1 |
| SmO_2_-*m.intercostales* (%) | 75±14 | 75±13 | 75±20 | 49±26 |  | 80±9 | 67±10 | 53±11 | 40±10 |
| ΔSmO_2_-*m.intercostales* (%) | - | - | - | 27±19 |  | - | - | - | 39±9^*^ |
| SmO_2_-*m.intercostales* (%-change) | 1.0 | 1.0±0.1**^#^** | 0.8±0.1**^#^** | 0.6±0.3 |  | 1.0 | 0.8±0.1 | 0.6±0.4 | 0.5±0.1 |
| SmO_2_-*m.vastus lateralis* (%) | 65±10 | 46±14 | 34±18 | 31±19 |  | 57±14 | 52±16 | 44±12 | 41±11 |
| ΔSmO_2_-*m.vastus lateralis* (%) | - | - | - | 34±18^*^ |  | - | - | - | 16±9^*^ |
| SmO_2_-*m.vastus lateralis* (%-change) | 1.0 | 0.7±0.2 | 0.5±0.2 | 0.5±0.3 |  | 1.0 | 0.9±0.2**^#^** | 0.8±0.1**^#^** | 0.7±0.1**^#^** |
| Ratio SmO_2_ (%) | 1.2±0.2 | 1.7±0.4 | 2.2±1.1**^#^** | 1.9±1.4**^#^** |  | 1.5±0.4 | 1.4±0.5 | 1.3±0.4 | 1.1±0.4 |
| Ratio SmO_2_ (%-change) | 1.0 | 1.5±0.4 | 2.0±1.0**^#^** | 1.8±1.2**^#^** |  | 1.0 | 0.9±0.2 | 0.8±0.2 | 0.7±0.2 |
| THb-*m.intercostales* (g·dL^-1^) | 12.5±0.4 | 12.4±0.5 | 12.3±0.5 | 12.2±0.7 |  | 12.0±0.3 | 11.9±0.3 | 11.8±0.3 | 11.8±0.3 |
| THb-*m.vastus lateralis* (g·dL^-1^) | 12.5±0.5 | 12.4±0.5 | 12.3±0.5 | 12.4±0.5 |  | 12.0±0.3 | 12.0±0.3 | 11.8±0.3 | 11.8±0.3 |
|  |  |  |  |  |  |  |  |  |  |

Data are presented as mean±standard deviation.

**#**: *p*<0.05 (two-way RM-ANOVA (sex × time), statistically difference between groups).

*****: *p*<0.05 (Student`s t-test, statistically difference between groups).

**Abbreviations**: VT1, first ventilatory threshold; VT2, second ventilatory threshold; WtW, workload-to-weight; $\dot{V}$O_2_, oxygen consumption; SmO_2_, muscles oxygen saturation; ΔSmO_2_, SmO_2_ rest – SmO_2_ peak phase; Ratio SmO_2_, SmO_2_-*m.intercostales*·SmO_2_-*m.vastus lateralis*^-1^; THb: total hemoglobin.
